# The new metrics of Anais Brasileiros de Dermatologia^؆^

**DOI:** 10.1016/j.abd.2026.501447

**Published:** 2026-08-03

**Authors:** Hiram Larangeira de Almeida, Jane Tomimori, Neusa Yuriko Sakai Valente, Renata Ferreira Magalhães

**Affiliations:** a Universidade Católica de Pelotas, Pelotas, RS, Brazil; b Universidade Federal de São Paulo, São Paulo, SP, Brazil; c Universidade de São Paulo, São Paulo, SP, Brazil; d Universidade Estadual de Campinas, Campinas, SP, Brazil

New metrics for scientific journals are traditionally released at the end of the first half of the year.

We have seen improvements in two key bibliometric indicators.

Our Impact Factor reached 4.4 (Fig. 1). It was calculated by dividing the citations received in 2025 for articles published in 2023 and 2024 by the total number of publications during those two years. Beyond the increase itself, when compared to other dermatology journals, we rank in the top 25% (quartile)—that is, in the Q1 category.

**Fig. 1:**
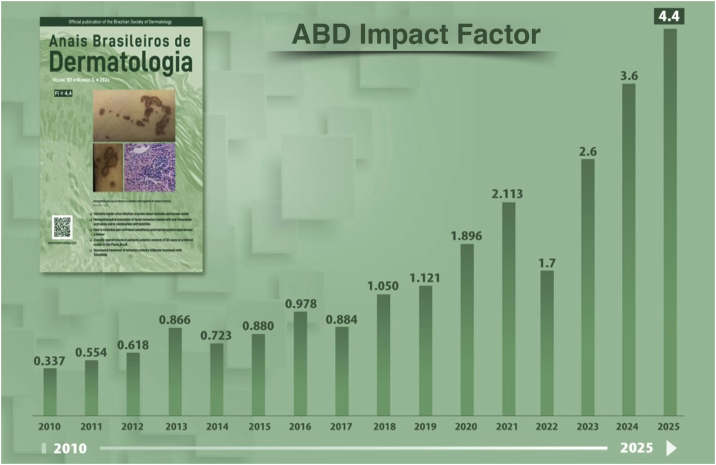
Evolution of the impact factor.

The other indicator, CiteScore, uses a longer analysis period. It divides citations over four years (in this case, 2022 to 2025) by the number of publications from that same period: we received 2136 citations over these four years and published 747 articles in the same period—meaning we are cited nearly three times as often as we publish—resulting in a CiteScore of 2.9 (Fig. 2).

**Fig. 2:**
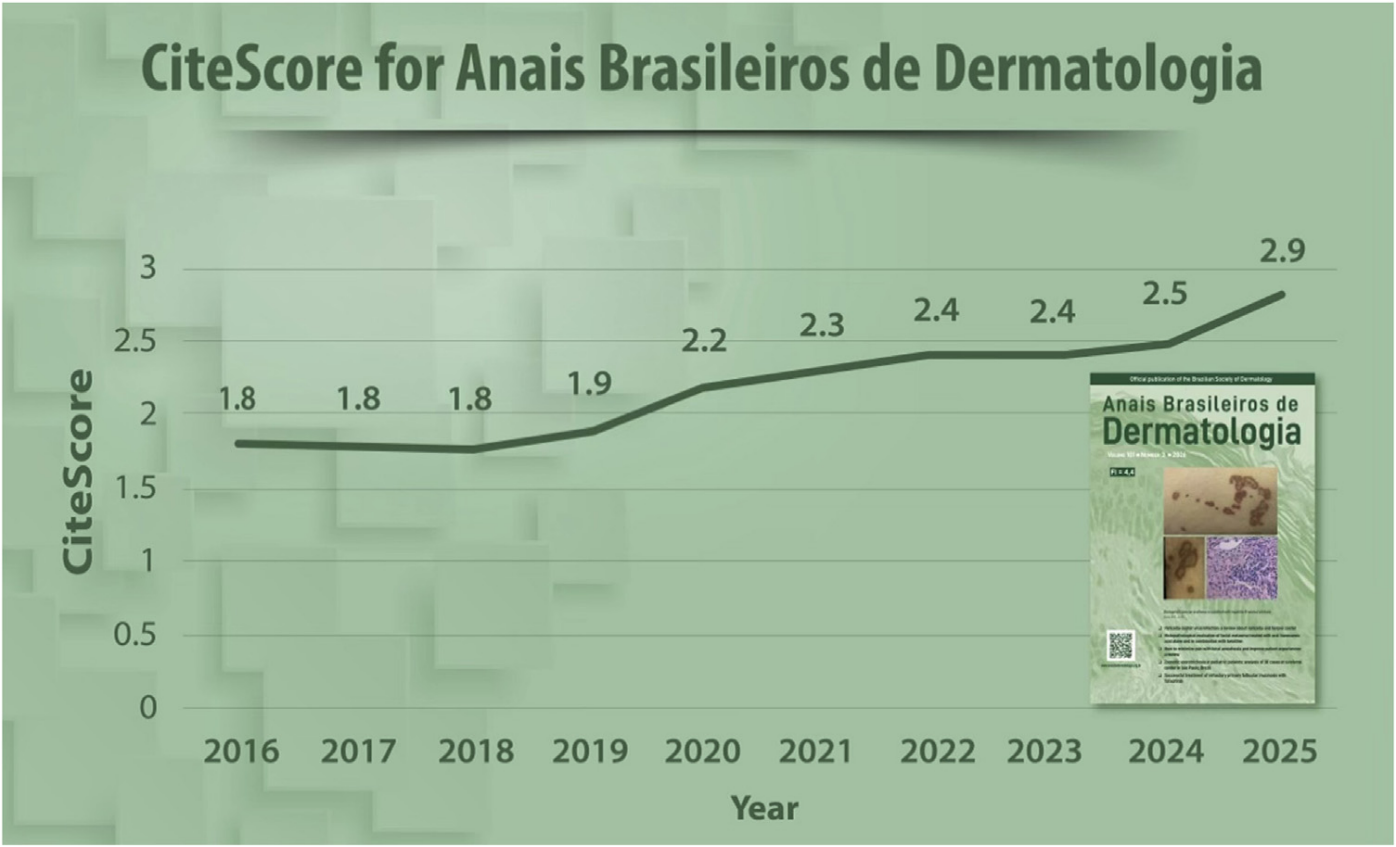
Evolution of CiteScore.

These results are the fruit of the work of the previous administration (2021–2025),^1^ characterized by rigor in the selection and editing of articles—an approach that is being continued by the current administration.^2^

In Greek, the word for price or esteem is *timi* (τιμή). With the addition of our familiar prefix *epi*-, the word *epistími* (επιστήμη) emerges [giving rise to the term "epistemology"]; *epistími* means "that which stands above value" and signifies SCIENCE, as it is inherently valuable.

We are convinced that we contribute to dermatological science by receiving submissions from around the world and publishing articles of relevance to the international community.

## Authors' contributions

Hiram Larangeira de Almeida Júnior: Approval of the final version of the manuscript; design and planning of the study; drafting and editing of the manuscript.

Jane Tomimori: Approval of the final version of the manuscript; design and planning of the study; drafting and editing of the manuscript.

Neusa Yuriko Sakai Valente: Approval of the final version of the manuscript; design and planning of the study; drafting and editing of the manuscript.

Renata Ferreira Magalhães: Approval of the final version of the manuscript; design and planning of the study; drafting and editing of the manuscript.

## Financial support

None declared.

## Research data availability

Does not apply.

## Declaration of competing interest

None declared.
